# Moxidectin: a review of chemistry, pharmacokinetics and use in horses

**DOI:** 10.1186/1756-3305-2-S2-S5

**Published:** 2009-09-25

**Authors:** Rami Cobb, Albert Boeckh

**Affiliations:** 1 Fort Dodge Animal Health, Pharmaceutical Research and Development, Princeton, NJ, USA

## Abstract

This article reviews the current knowledge of the use of moxidectin (MOX) in horses, including its mode of action, pharmacokinetic and pharmacodynamic properties, efficacy, safety and resistance profile.

Moxidectin is a second generation macrocyclic lactone (ML) with potent endectocide activity. It is used for parasite control in horses in an oral gel formulation. The principal mode of action of MOX and of other MLs is binding to gamma-aminobutyric (GABA) and glutamate-gated chloride channels. Moxidectin is different from other MLs in that it is a poor substrate for P-glycoproteins (P-gps) and therefore less susceptible to elimination from parasite cells through this mechanism. Due to its unique physicochemical and pharmacokinetic characteristics, MOX provides broad distribution into tissues, long half-life, significant residual antiparasitic activity, and high efficacy against encysted cyathostomin larvae. These characteristics allow for high efficacy and longer treatment interval against all important nematodes, when compared to other equine anthelmintics. A combination of MOX with praziquantel provides expanded spectrum of activity by adding activity against cestodes. Appropriate use of MOX allows for the development of strategic anthelmintic programmes that are different from those with conventional anthelmintics. Fewer treatments are required over a period of time, and therefore impose less frequent selection pressure for resistance.

## Introduction

Moxidectin is a potent, broad-spectrum endectocide with activity against a wide range of nematodes, insects and acari. It is used worldwide as a parasiticide in a variety of mammalian species including food-producing and companion animals.

The first MOX-containing product was an injectable formulation for cattle, approved for commercial use in Argentina in 1989. Subsequent formulations introduced worldwide for control of parasitosis include a tablet and a sustained release injectable for prevention of heartworm disease in dogs, injectable and oral drenches for sheep, a pour-on formulation for cattle and deer, and sustained release injectable formulations for cattle and sheep. These long-acting formulations provided significantly longer persistent activity than the earlier formulations, including season-long control against some parasite species.

Combination products, including an injectable formulation of MOX with clostridial and caseous lymphadenitis vaccines for sheep, are commercially available in major sheep producing countries. Additionally, an ongoing collaboration between Wyeth/Fort Dodge Animal Health and the World Health Organization led to the development of an oral formulation of MOX for humans, which is being evaluated in several African countries for prevention of river blindness, a parasitic disease caused by *Onchocerca volvulus*.

For horses, MOX is formulated as unique, easy-to-administer oral gel formulation that provides excellent and long-lasting efficacy against nematodes and gastrointestinal bots. A second gel formulation containing MOX in combination with praziquantel adds efficacy against cestodes.

## Chemistry and pharmacology

Moxidectin is a semi-synthetic methoxime derivative of LL F-2924α, commonly referred as F-alpha or nemadectin, a 16-member pentacyclic lactone of the milbemycin class. F-alpha is a product of fermentation of *Streptomyces cyaneogriseus *subsp. *noncyanogenus*, a bacterial organism isolated in 1983 from a sample of sand from Victoria, Australia. F-alpha possesses strong anthelmintic activity but has limited ectoparasiticide activity. Moxidectin is the result of chemical optimization of F-alpha. Moxidectin differs from ivermectin (IVM) by the absence of a dissacharide moiety on carbon-13, a substituted olefinic side chain at carbon 25 and a unique methoxime moiety at carbon-23. Due to differences in chemistry and biological activity compared to other MLs developed for similar uses, MOX is classified as a second generation ML.

Studies to determine the mode of action of MLs on parasites demonstrated that these compounds act by binding to ligand-gated chloride channels, more specifically the subtypes that are gamma-aminobutyric (GABAA) mediated and glutamate-gated [1]. The consequence of ML binding and activation is an increased permeability, leading to an influx of chloride ions and flaccid paralysis of the parasite leading to death. Recent work identified a dopamine-gated ion channel (HcGGR3) in *Haemonchus contortus *that is associated with ML resistance [2].

A number of differences in the modes of action of avermectins and milbemycins have been proposed for ruminant parasites. One example is the difference in the degree of pharyngeal pumping activity present in *Haemonchus contortus *that has undergone selection with IVM. In this selected strain of *H. contortus*, pharyngeal pumping activity is altered in presence of IVM, but unchanged in the presence of MOX, suggesting to some extent a difference in mode of action [3]. Another example is the degree of transport of MLs by p-glycoproteins (P-gp). P-gps are transmembrane pumps that have been shown to reduce the uptake of lipophilic compounds from the GI tract, to limit penetration into tissues, including brain, and to enhance elimination by biliary, intestinal or renal secretion. MOX is a poor substrate for P-gps, compared to other MLs, such as IVM and selamectin. This is demonstrated by the significantly lower rate (p < 0.01) of transport of radiolabeled MOX, compared to radiolabeled IVM and selamectin, across human intestinal epithelial cell monolayers and canine peripheral lymphocyte membrane both from basal to apical direction as well as from apical to basal direction [4].

The toxicity of MLs is believed to be dependent, in part, on transport by P-gps [4,5]. P-glycoproteins act as transport proteins able to carry certain drugs, including MLs, across cell membranes. Over- or under-expression of P-gps can lead to accumulation or depletion of a compound within cells.

In the case of over-accumulation in cells of the nervous system, signs of neurotoxicity may be seen, even when the recommended commercial dose has been administered. Investigations into the sensitivity of some collie-type dogs to IVM have identified the presence of genetically based P-gp deficiency which leads to neurotoxicity at doses considered safe in the broader canine population. Comparisons of the affinity of MLs for P-gps demonstrated that MOX is a poor substrate for P-gps and differs in this property by an order of magnitude from the avermectins, such as IVM. This may explain the greater mammalian safety of MOX compared to IVM, most notably in P-gp deficient animals, such as some collie dogs [6-8]. This property of MOX means the benefits of monthly, or longer sustained release protection against heartworm infection is available for dogs susceptible to toxicity from IVM-based products [9].

The converse of P-gp deficiency, namely P-gp over-expression, is increasingly identified as being an important factor in anthelmintic resistance in nematodes. A clinical conundrum for many years has been why MOX is effective in controlling IVM-resistant nematodes when both are classified as MLs. The differing affinity for P-gps of MOX in comparison with the avermectins may play an important role. Selection on a P-gp gene has been identified in ML-selected nematode strains [10]. However, quantitative and qualitative differences have been identified between MOX and IVM in this selection. Further studies of the P-gps lend additional support to the importance of these transport proteins in the development of resistance to MLs [11,12].

The disposition of MOX in horses is well described, including its metabolic fate. Studies conducted with the use of radiolabeled MOX formulated as an oral gel demonstrated that fecal excretion of the parent compound was the main elimination pathway, accounting for 77% of the dose administered [13], thus confirming that the product remains predominantly as the active parent compound and is not extensively broken down to less active metabolites.

Comparative pharmacokinetics of MOX and IVM after oral administration to horses were also performed [14]. Horses were treated orally with a gel formulation at the label dose of 0.4 mg of MOX per kg of body weight. Blood was collected over time and plasma assayed by high performance liquid chromatography with fluorescence detection. Results showed MOX has a favorable pharmacokinetic profile compared to IVM, including a longer elimination half-life and greater area under the curve. These are summarized in Table 1. The longer time course of MOX in horses leads to a longer exposure of parasites to MOX. This delivers a positive impact on efficacy as demonstrated by the longer egg reappearance interval following treatment and the high efficacy against stages of parasites not readily controlled by other compounds. In addition to plasma pharmacokinetics, another study documented the residue profile of MOX in selected horse tissues after oral administration of the commercial gel formulation at the label dose [15]. Concentrations of MOX in tissues are summarized in Table 2. As expected, due to its lipid solubility, MOX was found at highest levels in the abdominal and subcutaneous fat. This fat depot naturally releases MOX into the systemic circulation over time and contributes to the long half-life and sustained activity. Conversely, little MOX was found in lean tissues, such as muscle, supporting a short withholding period for meat. This is consistent with tissue residue data from cattle [16] and sheep [17] confirming a consistent metabolic and residue profile for MOX in major domestic species.

**Table 1:** Comparative pharmacokinetics of moxidectin (MOX) and ivermectin (IVM) after oral administration in horses expressed as mean (± SD)

	**IVM**	**MOX**
Dose (mg/kg)	0.2	0.4
Cmax (μg/mL)	44.0 (± 23.1)	70.4 (± 10.7)
Tmax (day)	0.38 (± 0.24)	0.37 (± 0.19)
T1/2 elim (day)	4.25 (± 0.29)	23.11 (± 11.0)
AUC 0-t (μg/day/mL)	132.7 (± 47.3)	363.6 (± 66.0)

Cmax = Maximum concentration

Tmax = Time for maximum concentration

T1/2 = Terminal half-life

AUC = Area Under the concentration vs. time Curve

**Table 2:** Tissue concentrations of moxidectin (MOX) after oral administration of 0.4 mg/kg to horses.

**Tissue**	**Concentration of MOX (ppb)**
Abdominal fat	884
Back fat	664
Liver	184
Kidney	51
Muscle	21

ppb = parts per billion

## Efficacy and safety

The efficacy of MOX for nematode control in horses is well documented in controlled laboratory studies, field efficacy studies and by years of use by veterinarians and horse owners around the world. Typical study results are reported by Cleale et al. in a multicenter evaluation of pastured horses in 3 states of the United States, where 72 pastured horses were studied in Idaho, Illinois and Tennessee [18]. Animals had an average age of 32, 32 and 18 months in Idaho, Illinois and Tennessee, respectively and average pre-treatment fecal egg counts (EPG) of 266 (range 78-392), 108 (range 31-1042) and 576 (range 10-2850) eggs in the same States. The horses were randomly divided into 2 groups, one treated with MOX gel at the label dose of 0.4 mg/kg orally and the other group left as untreated control. Parasiticidal efficacy was determined by necropsy and parasite count, conducted 12 to 14 days after treatment. Horses were determined to have a mixed infection of ascarids, small strongyles, large strongyles, tapeworms and bots. As expected, no efficacy of MOX was observed against the tapeworm *Anoplocephala perfoliata*. For all other parasites encountered, including *Gasterophilus *spp. larvae, adult and larval stages of ascarids and large and small strongyles, efficacy was greater than 95%, representing a significant difference from control (p < 0.05) as demonstrated in Tables 3 and 4, which contains pooled data from all 3 geographical locations.

**Table 3:** Geometric means of egg counts and percent efficacy of moxidectin (MOX) against non-cyathostomins, compared to control

	**Geometric means**		
	
	**control**	**MOX**	**Percent Efficacy**
*Anoplocephala perfoliata*	4.56	2.58	43.3
*Gasterophilus intestinalis *2nd instar	16.19	0.66*	95.5
*Gasterophilus intestinalis *3rd instar	68.1	2.75*	96.0
*Gasterophilus nasalis *2nd instar	2.24	0.00*	100.0
*Gasterophilus nasalis *3rd instar	5.27	0.02*	99.6
*Oxyuris equi *L4	8.39	0.31*	96.3
*Parascaris equorum *adult	1.78	0.07*	96.3
*Parascaris equorum *L4	1.02	0.00*	100.0
*Strongylus edentatus *adults	2.41	0.03*	98.7
*Strongylus vulgaris *adults	5.27	0.21*	96.1
*Triodontophorus brevicaudata *adults	9.49	0.00*	100.0
*Triodontophorus serratus *adults	22.04	0.00*	100.0

* significant difference from control (p < 0.05)

**Table 4:** Geometric means of egg counts and percent efficacy of moxidectin (MOX) against cyathostomins, compared to control

	**Geometric means**		
	
	**Control**	**MOX**	**Percent Efficacy**
*Coronocyclus coronatus*	205.06	0.07*	99.97
*Coronocyclus labiatus*	126.98	0.00*	100
*Coronocyclus labratus*	403.12	0.09*	99.98
*Cyathostomum catinatum*	3917.4	0.00*	100
*Cyathostomum pateratum*	128.89	0.00*	100
*Cylicocyclus brevicapsulatus*	9.41	0.00*	100
*Cylicocyclus elongatus*	8.59	0.00*	100
*Cylicocyclus insigne*	134.65	0.18*	99.86
*Cylicocyclus leptostomum*	935.71	0.00*	100
*Cylicocyclus nassatus*	5073.92	0.09*	>99.99
*Cylicocyclus radiatus*	46.45	0.09*	99.81
*Cylicostephanus calicatus*	1080.56	0.09*	99.99
*Cylicostephanus goldi*	113.53	0.00*	100
*Cylicostephanus longibursatus*	7518.56	0.09*	>99.99
*Cylicostephanus minutus*	5585.9	0.66*	99.99
*Petrovinema poculatus*	18.89	0.00*	100
*Poteriostomum imparidentatum*	3.69	0.00*	100
*Luminal cyathostomin*L4	3726.11	21.60*	99.42

* significant difference from control (p < 0.05)

Other efficacy studies have demonstrated differences in results following the treatment of cyathostomes by either MOX or IVM. One such study was conducted on six horse farms in Northwest Arkansas [19]. A total of 96 horses that were scheduled for anthelmintic treatment were randomly divided into 3 groups and treated with either MOX (QUEST - Fort Dodge Animal Health, Overland Park, KS, USA), IVM (EQVALAN - Merial Limited, Duluth, GA, USA) or fenbendazole (FBZ) (PANACUR - Intervet/Shering-Plough, Millsboro, DE, USA), according to label directions. Evaluation of efficacy was performed by fecal egg count tests comparing counts prior to treatment, on Day 0, with FECs conducted on Days 56, 84 and 112 after treatment. On Day 0, FECs did not vary across treatment groups, but significant differences were seen across treatment groups on the post-treatment evaluations, with superior efficacy of MOX, followed IVM and FBZ (p < 0.05), as demonstrated in Table 5. The results of this study have great significance in the development of strategic anthelmintic dosing programmes in that they not only confirm the high efficacy of MOX against key equine parasites, but also show that less frequent anthelmintic treatments are needed if MOX is used for cyathostome control.

**Table 5:** Percent reduction in nematode egg count after treatment on Day 0

	**Days after treatment**		
	
	**56**	**84**	**112**
Moxidectin	99.1	97.6	94.9
Ivermectin	85.9	24.2	-8.1
Fenbendazole	16.4	-27.0	-32.0

While MOX may be used interchangeably with other effective anthelmintics for broad spectrum control of nematodes, it also has the potential to be used differently by applying its known characteristics of high potency and long duration of activity. Sustainable worm control programmes can be designed that provide the same level of protection from worms through fewer treatments, thus reducing the selection pressure for resistance.

One of the significant differentiating factors of MOX from other MLs is the efficacy against encysted stages of cyathostomes, the causal agents of larval cyathostominosis. This clinical syndrome is characterized by the simultaneous emergence of larvae leading to significant inflammatory enteropathy in the cecum and colon, and resulting in colitis, weight loss, diarrhea and colic. The high efficacy of MOX against inhibited stages of cyathostomins is well documented in the published literature [20-22]. A dilemma for clinicians in managing control of cyathostomin infections is whether treatment with an anthelmintic effective against luminal and developing stages only, will trigger mass emergence of inhibited stages with the associated clinical syndrome, or whether treatment using larvicidal products will lead to a mass die off of inhibited stages that may cause inflammatory responses in the mucosa and colic. A recently published study described the evaluation of the degree of inflammation caused by the killing of encysted cyathostomins larvae using either MOX or FBZ [23]. In that study, MOX was administered orally once at 0.4 mg/kg, while FBZ was administered daily for 5 days at the dose of 7.5 mg/kg. Animals were then sacrificed and parasites recovered 14 days after treatment. While both compounds were effective in removing larval cyathostomins, indicating a strain that is susceptible to both compounds, there was a significant difference in the degree of mucosal inflammation associated with the larval kill. Histologically, in animals treated with FBZ, T-lymphocytes accumulated around the intact larvae forming a granuloma along with eosinophils, with the inflammation extending into the mucosa and being associated with ulcerations. Conversely, in horses treated with MOX, morphological alterations were not observed in histopathology, and the disintegrated larvae resorbed without causing severe inflammation in the gut wall. Based on the safety and efficacy profile demonstrated for MOX, the clinician has available the means to control all stages of cyathostomins without the necessity to adjust from a normal dose or to administer repeated treatments, and without inducing severe inflammatory responses.

The use of MOX in pregnant mares was demonstrated to be safe in studies conducted during the registration phase of the gel formulation [24]. In addition a study was conducted to explore the impact of a strategic MOX treatment of perinatal mares on worm control in their foals [25]. In that study, a group of 25 pregnant mares was divided into 2 groups with 12 being treated with MOX oral gel at the label dose around the time of foaling, and 13 were left as untreated controls. Foals were born from Day -1 to Day 76. Based on coproculture and pasture analysis, the nematode challenge was predominantly of *Cyathostomum *spp. Complete control of parasites, including *Strongyloides westeri*, was maintained for at least 91 days in the mares and in the foals born from mares that were treated with MOX. In this study, the parasite challenge in the mares was significant, and the control mares had to be treated with IVM on grounds of welfare. At the end of the grazing period, the foals born from mares treated with MOX had an average body weight 17 kg higher than those born from mares that were treated with IVM (control mares) despite the fact that their foals were themselves treated with IVM at the age of 3 months. The potential of using MOX in perinatal mares to control nematode infections in foals warrants further investigation.

The potential of tapeworms to induce colic in horses has been documented [26-28] and combining MOX with the potent cestocide praziquantel allows simultaneous treatment of roundworms and tapeworms in a single application. The efficacy and safety of this combination product was demonstrated in a large field evaluation where four hundred client-owned horses were treated with either the combination of MOX + praziquantel or control. In that study, no adverse event was observed and a reduction of over 99% of *Anoplocephala *spp. and over 98% reduction in strongyle egg counts was observed [29]. Other less prevalent tapeworms, such as *Anoplocephala magna *and *Paranoplocephala mamillana*, are also known to have clinical significance in the horse [30]. Efficacy studies conducted on horses naturally infected with cestodes that were divided into matching treated and untreated groups demonstrated, at necropsy, the complete efficacy of the combination of MOX plus praziquantel against these tapeworms [31].

Another differentiating feature of MOX compared to other MLs is the environmental profile, most notably the low impact of MOX residues in faeces on the emergence rate of dung beetles in contrast to the findings with avermectins [32]. This was demonstrated in evaluation of the rate of emergence of the dung beetle *Aphodius constans *exposed to feces of horses treated orally with the combination of MOX and praziquantel [33]. Dung beetles play an important role in parasite control by providing rapid dispersal and/or burial of dung and therefore reducing the habitat for nematode eggs and larvae, in addition to providing improvement in pasture.

Concerns about the potential development of resistance to MLs have been debated in the equine parasitology community for some years [34,35]. Increasing concerns have arisen with recent reports of the inefficacy of MLs, both IVM and MOX against *Parascaris equorum*. Such failures were reported initially from the Netherlands [36], later from North America [37-39], then other European countries [40-42] and most recently Brazil [43]. Frequency of anthelmintic treatments has been cited as a major contributing factor for selection for resistance by many parasitologists, and a review of common nematode control practices in horses indicates the highest frequency of treatments is in foals, specifically for the control of ascarids. Hence, the appearance of resistance under these conditions is not surprising, also taking into account that the initial efficacies of MLs against *P. equorum *indicated this was a dose-limiting species for this group of compounds. The reduced efficacy of anthelmintics in young versus adult horses has been long recognized [44]. Further concerns on the development of ML resistance are related to the reported reduction in the egg reappearance period (ERP) of cyathostomins following IVM treatment [45]. This is taken as a first sign of reduced sensitivity of cyathostomins to IVM.

The complexity of the mechanisms of resistance to MLs and our incomplete understanding of them has led to varied and different proposals for the best way of minimizing selection for resistance and preserving the efficacy of MLs for as long as possible. Factors to consider include optimal treatment frequency; should the less potent compound, IVM, be used until it is ineffective, then MOX used to control these parasites; what role as "refugia" is played by inhibited stages that are not effectively controlled by IVM, but against which MOX has high potency; and is there a link between benzimidazole resistance and ML resistance as selection has been shown at the beta-tubulin site for both classes of compounds [46].

One aspect on which there is general agreement is that anthelmintic treatments should be used only when needed, and recommendations have been made to monitor faecal egg counts and treat individual horses only when needed. A consensus on the appropriate threshold for treatment has not been reached but is of the order of 150-200 eggs per gram. The extended egg reappearance interval for MOX means the same level of control can be obtained with fewer treatments than with IVM [47-49]. The issue of the impact of treating inhibited cyathostomes has led to heated debate. The point is moot if horses are treated in the absence of these inhibited stages. Ivermectin does not deliver effective control of these stages [50,51] even at elevated doses [52], however efficacies of the order of 35% to 77% [53] have been reported. Therefore, characterizing such populations as "unexposed and unselected refugia" is both simplistic and incorrect, survivors of the exposure to IVM are able to mature and contribute eggs to the next generation. In contrast, the high efficacy of MOX against all mucosal stages [22,54] leaves few survivors and the contribution of resistance alleles to the next generation is much reduced. Taking into account the current knowledge of rates of selection for resistance in nematodes [55] and the quantitative and qualitative differences in mechanisms of action of resistance, it is strongly recommended that MOX be used strategically as a first line treatment in equine nematode control programmes, and not "saved" for dealing with IVM resistant strains. Although MOX has been shown to control many IVM resistant parasites once resistance to therapeutic levels of IVM has been recognized [56-59] it is likely that the first steps have been taken towards MOX resistance [60-62]. Research continues to elucidate the similarities and differences in IVM and MOX resistance, particularly in relation to P-gps, Multi-drug Resistance Proteins (MRPs) and ABC Transporters [3-5,10-12]. "This new data in a parasitic nematode confirms previous evidence obtained in *C. elegans *that MOX has markedly different and less effect on causing overexpression of both P-gps and MRPs in nematodes compared with IVM. As P-gps and MRPs are involved in the efflux of xenobiotics, their overexpression appears to be a major mechanism of IVM resistance that is not shared, or not shared to the same extent by MOX selection." [63]

Specifically in relation to equines, the presence of at least two P-gp genes has been confirmed in PCR studies conducted in nine different species of cyathostomins [64].

The best uses of MOX will be strategic and pre-emptive use before any ML resistance.

## Conclusion

Moxidectin provides safe and effective means of parasite control for horses. As a second-generation ML, it has different chemical, pharmacokinetic and pharmacodynamic characteristics than the first generation MLs, the avermectins, such as higher potency, longer half-life and better diffusion into relevant tissues such as intestinal mucosa. Moxidectin has the longest 'egg reappearance period' after treatment, requiring fewer treatments over a period of time for the same level of control of parasites. This is important, as fewer treatments represent less opportunities for development of nematode resistance, as selection pressure is applied fewer times, compared to other anthelmintic treatments. Through the application of the known scientific differences unique to MOX we can develop strategic worm control programmes different from those with conventional anthelmintics. An example is the perinatal treatment of mares that can be a viable option to reduce worm transmission to their foals, and to prevent the buildup of infective larvae on foaling pastures. Moxidectin can be used as a conventional anthelmintic in horses but also provides great flexibility and convenience for treatment of important parasitosis in horses, regardless of age or pregnancy status.

## Competing interests

The authors acknowledge their affiliation with Fort Dodge Animal Health, the manufacturer of moxidectin, and declare no conflict when writing this review.

## Authors' contributions

The two authors contributed equally in the preparation of this review.
